# Dual role of GRK5 in cancer development and progression

**Published:** 2016-05-16

**Authors:** J Gambardella, A Franco, C Del Giudice, A Fiordelisi, E Cipolletta, M Ciccarelli, B Trimarco, G Iaccarino, D Sorriento

**Affiliations:** 1 Department of Medicine and Surgery -University of Salerno, Italy;; 2 Department of Advanced Biomedical Science -“Federico II” University of Naples, Italy;; 3 Institute of Biostructure and Bioimaging - CNR, Naples, Italy.

**Keywords:** GRK5, GPCR, cancer

## Abstract

GRK5 is a multifunctional protein that is able to move within the cell in response to various stimuli to regulate key intracellular signaling from receptor activation, on plasmamembrane, to gene transcription, in the nucleus. Thus, GRK5 is involved in the development and progression of several pathological conditions including cancer. Several reports underline the involvement of GRK5 in the regulation of tumor growth even if they appear controversial. Indeed, depending on its subcellular localization and on the type of cancer, GRK5 is able to both inhibit cancer progression, through the desensitization of GPCR and non GPCR-receptors (TSH, PGE2R, PDGFR), and induce tumor growth, acting on non-receptor substrates (p53, AUKA and NPM1). All these findings suggest that targeting GRK5 could be an useful anti-cancer strategy, for specific tumor types. In this review, we will discuss the different effects of this kinase in the induction and progression of tumorigenesis, the molecular mechanisms by which GRK5 exerts its effects, and the potential therapeutic strategies to modulate them.

## I. INTRODUCTION

GRKs (G-protein-coupled- receptor kinases) are a family of serine/threonine kinases traditionally known for their ability to recognize and phosphorylate agonist-activated G-protein-coupled receptors (GPCRs), leading to their desensitization [1]. In particular, the agonist-dependent conformational change of the receptor renders the latter available for GRK-mediated-phosphorylation, leading to G-protein-uncoupling, to the increase of GPCR affinity for arrestins and clathrin-dependent receptor internalization [2]. In this manner, GRKs act as crucial negative regulators of a variety of GPCRs, including adrenergic receptors, muscarinic receptors, dopamine, opioid and chemokine receptors [3–6]. Seven subtypes of GRKs (GRK1–7) have been identified to date, subdivided into three groups depending on sequence homology: rhodopsin kinases or visual GRK subfamily (GRK1 and GRK7), the β-adrenergic receptor kinases subfamily (GRK2/GRK3) and the GRK4 subfamily (GRK4, GRK5 and GRK6) [1, 7, 8]. GRKs share a common basic structural architecture [1], characterized by a well-conserved central catalytic domain (∼270 aa), a variable-length carboxyl-terminal domain (∼105–230 aa) and an N-terminal domain (∼185 aa) which includes a Regulator of G protein Signaling Homology (RH) domain [8, 9]. Several studies demonstrate that the expression and activity of GRKs are impaired in many pathological conditions [10–12]. Although GRKs are highly selective for agonist activated GPCRs, others substrates have been identified in the last decade. Indeed, GRKs are able to phosphorylate non-GPCR receptors, such as PDGF-receptor [13], and non–receptor substrates, such as tubulin, synucleins, the β-subunit of the epithelial Na+ channel, insulin receptor substrate 1 (IRS-1), NF-kB inhibitor (IkBα), and others soluble substrates [14–18]. Moreover, GRKs regulate intracellular signaling also in a phosphorylation-independent manner, through the direct interaction with a variety of proteins involved in cellular signaling and trafficking [19–21]. Thus GRKs are multifunctional proteins able to regulate key cellular processes, from receptor activation to nuclear transcription [22], in different cellular compartments [23–25], due to their ability to move within the cell in response to stimuli [25, 26].

## II. G-PROTEIN COUPLED RECEPTOR KINASE 5

Among GRKs, GRK5 is one of the most studied since it is involved in several pathologic conditions [27–29]. Indeed, GRK5 is up-regulated in failing human myocardium [29–31], and its over-expression in the heart reduced cardiac β-AR responsiveness [32, 33]. Several reports underline the involvement of GRK5 in the regulation of glucose metabolism [30, 34, 35]. Indeed, it has been shown that, the overexpression of GRK5 enhances the internalization of Glucagon Receptor, a GPCR that mediates hyperglycaemic effects of glucagon in Diabetes [36, 37]. Accordingly, GRK5 gene deletion in mice, leads to impaired glucose tolerance and insulin sensitivity [30]. GRK5 attenuates atherosclerosis through multiple cell type-specific mechanisms including desensitization of Tyrosine Kinase receptors, such as macrophage CSF-1R, and the SMC platelet-derived growth factor receptor-β (PDGFR) [28]. Several reports show that GRK5 is also involved in Parkinson disease, due to its ability to phosphorylate the α-synuclein, modulating dopamine-up-take [38, 39].

GRK5 effects on the regulation of intracellular signaling depend on cell type, stimuli, and kinase localization within the cell. GRK5 binds to cellular membranes through C-terminal polybasic domain and an N-terminal phosphatidyl- inositol -4-5-bisphosphate (PIP2) binding site [40, 41]. While GRK2 and GRK3 are primarily cytoplasmic and are targeted to plasma membrane by binding Gβγ subunits through their C-terminal pleckstrin homology domain, GRK5 constitutively binds phospholipids and displays preferential membrane localization [19]. However, GRK5 is also able to localize into the nucleus, through means of functional nuclear localization sequence (NLS), that allows the kinase to enter the nucleus and bind DNA in vitro [24]. Such nuclear localization is regulated by intracellular calcium levels. Indeed, the activation of M3MR increases intracellular calcium levels and activates the calcium sensor protein, CaM, which on turn binds the N-terminal domain of GRK5 promoting its nuclear export [24].

GRK5 shuttles between the cytosol and the nucleus and regulates the activities of transcription factors at different levels. Indeed, GRK5 phosphorylates histone deacetylase 5 (HDAC5), a repressor of myocyte enhancer factor 2 (MEF2), leading to nuclear export of HDAC5 and activation of MEF2 transcription activity, in cardiac hypertrophy [42]. Moreover, in a model of Parkinson disease, the nuclear accumulation of GRK5 inhibits gene transcription of apoptosis regulator bcl-2, probably increasing HDAC activity [43].

Moreover, GRK5 regulates the activity of the transcription factor NFκB [44]. Since it binds the inhibitory protein of NFκB, IκBα, by means of the RH domain (GRK5-RH) and stabilize the complex IκBα/NFκB in the nucleus, thus inhibiting NFκB transcriptional activity. The intracardiac injection of an adenovirus encoding the N-terminal domain of GRK5, AdGRK5-NT, reduces left ventricular hypertrophy by inhibiting NFκB-dependent hypertrophic gene expression [45]. Furthermore, Hullmann et al. showed that GRK5, acting in a kinase independent manner, is a facilitator of NFAT activity [46]. All this findings suggest that GRK5, trafficking within the cell, regulates key cellular processes that are involved in the development and progression of several pathological conditions.

GRK5, in addition to the above described roles in the cytoplasm and recently discovered activities in the nucleus, is now also reported herein to be functionally localized to centrosomes [47]. The localization of this kinase within the centrosome suggests another way by which GRKs can affect cellular processes.

## III. ROLE OF GRK5 IN CANCER

It’s known that the oncogenic phenotype derives from diverse genetic alterations that cause constitutive activation or a loss of function of proteins, such as the loss of allelic heterozygosity [48, 49]. Importantly, chromosome translocation of the 10q24 region has been specifically observed in several tumors, such as thyroid adenoma and glioma [50], suggesting that alteration of some genes in this region could affect the development of tumors. Since GRK5 maps on chromosome 10, at q24 region [51], it is likely that alteration of GRK5 gene, could be involved in certain thyroid or glia tumors. Several reports underline the involvement of GRK5 in the regulation of tumor growth. However, the effectS of GRK5 on cancer cell proliferation are controversial depending on its subcellular localization and on the type of cancer. In this review, we discuss these different effects of GRK5 on tumor progression, and the different molecular mechanisms by which GRK5 exerts these effects. Moreover, we will debate about the potential therapeutic strategies for cancer focused on GRK5 as main target.

### A. GRK5 inhibits tumor growth by regulating GPCR signaling

**Figure 1:**
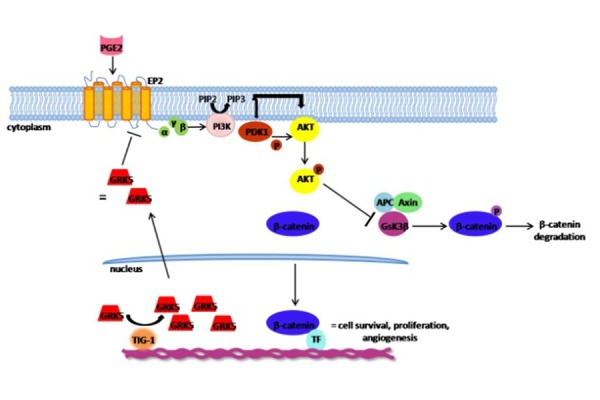
TIG-1 inhibits PGE2-dependent cell proliferation through the induction of GRK5 expression

G protein-coupled receptors (GPCRs), the largest family of cell surface proteins involved in signal transduction, have recently emerged as key regulators of tumor growth and metastasis [52]. Their role in the tumorigenic process depends on the specific tumor type and is due to their direct interaction or cross-talk with others receptors (i.e. EGF receptors) [52–54]. Thus, the pharmacological manipulation of these receptors is becoming an attractive goal for the development of novel strategies to target tumor progression and metastasis. In this context, it is not surprising that GRK5, could be a molecular target of these novel therapeutic strategies. Several studies show that GRK5 may be a negative regulator of cancer cell proliferation, mainly through its ability to desensitize GPCR on plasma membrane. Tsai et al. have shown that in colon cancer cells HCT116, the expression of Tazarotene-induced gene 1 (TIG-1), a putative tumor suppressor, inhibits cell proliferation by inducing GRK5 expression in response to PGE2 [55, 56]. TIG1 suppresses PGE2-stimulated cell proliferation through inhibition of β-catenin pathway, and this effect is mediated by GRK5, since it is ameliorated by GRK5 siRNA. Moreover, the over-expression of GRK5 suppressed PGE2-stimulated β-catenin activation in a dose dependent manner in HCT116 and DLD-1 cells (Fig. 1).

Also in thyroid cancer cells, GRK5 has negative effects on tumor growth, due to its ability to down-regulate GPCRs activity, in particular TSH-receptor activity [57]. The TSH receptor, is a major determinant of thyroid function and most of PGE2 binds and stimulates EP2, a GPCR, that activates PI3K; PI3K converts PIP2 in PIP3, that recruits PDK1 and AKT on plasmamembrane. PDK1 phosphorylates and activates AKT, that inhibits GSK3β, blocking β-catenin degradation and promoting cell proliferation.

TIG-1 induces GRK5 over-expression which on turn inhibits EP2, blocking its signaling transduction with an inhibitory effect on cell proliferation.

the TSH effects on proliferation and differentiation of thyroid cells are mediated by cAMP via an adenylyl- cyclase-activating Gs protein [58]. Indeed, several thyroid cancer types (DTC) have high levels of cAMP compared with normal thyroid tissue (NTT), and it has been demonstrated that this is associated with a reduction of the expression of GRK5 gene and protein levels. The data suggest that GRK5 is involved in the regulation process of TSH-stimulated cAMP response in human DTC. Thus, it is likely to speculate that GRK5 down-regulation of GPCRs could be one of the mechanisms that ensure a proliferative advantage to cancer cell, because the loss of GRK5 activity, allows the cell to escape to a control mechanism of the cellular growth.

**Figure 2.**
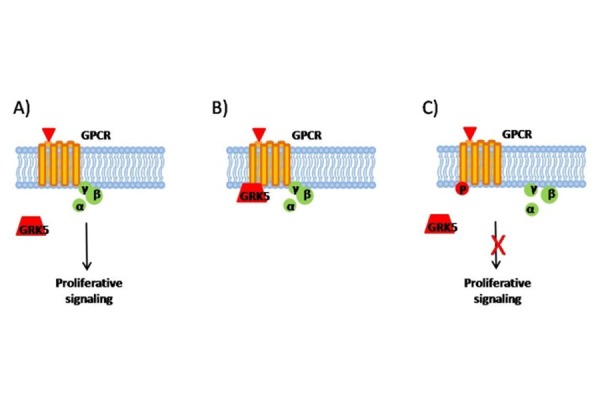
GRK5 inhibits tumor growth through phosphorylation of GPCR A) GPCRs are activated through the interaction with a specific ligand. The activated receptor, through G-protein, stimulates proliferative signaling. B–C) GRK5 interacts (B) and phosphorylates (C) GPCR, promoting un-coupling of G-protein and inhibition of proliferative signaling.

GRK5 inhibits cancer cell proliferation in the Kaposi’s sarcoma [59]. In tissues of patients with Kaposi’s sarcoma, human herpesvirus -8 (KSHV) is consistently present. This gamma-herpesvirus may be involved in the pathogenesis of primary effusion (or body cavity-based) lymphomas [60] and Kaposi’s sarcoma (KS) [61], and it contains a gene that encodes a G protein–coupled receptor (KSHVGPCR) [62]. KSHV-GPCR is constitutively activated and its expression stimulates cell proliferation and causes transformation of mouse fibroblasts [63, 64]. The co-expression of GRK5 inhibits KSHV-GPCR–induced cell proliferation and prevents transformation of rodent fibroblasts. Altogether, these findings support the proof of concept that GRK5 inhibits tumor growth in different cancer cells, through the phosphorylation of GPCR (Fig. 2) on plasmamembrane.

### B. Non-GPCR receptors and tumor growth

Besides GPCRs, other receptor signaling are involved in the development of cancer, such as Thirosine-kinase receptors (TRK) and Frizzled family receptors [65, 66]. It is known that the activation of these receptors is regulated by GRK5 in different cell types [67, 68], although it was never explored in cancer cells. Thus, it is likely that GRK5 could regulate tumor growth also through the regulation of TRK and Frizzled receptors. This could be the inspiration for future studies.

### C. GRK5 promotes tumor growth

Besides the above described inhibitory effect of GRK5 on tumor growth when it is located on plasma membrane, in other compartments GRK5 interacts with several intracellular molecules and modulates their stability and activity, thus favoring tumor development and progression. Here we report the effect of GRK5 on some of these molecules.

### D. GRK5 regulates p53

**Figure 3.**
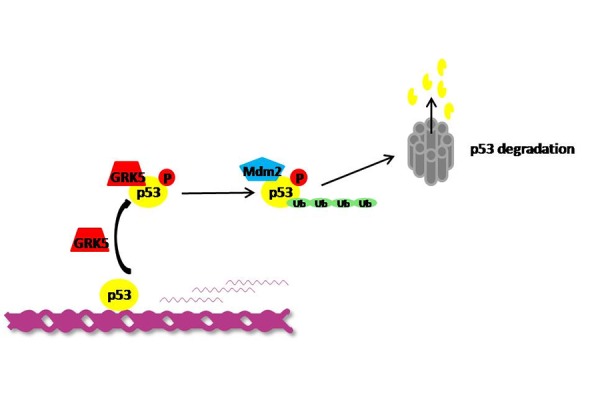
GRK5 promotes p53 degradation GRK5 interacts with and phosphorylates p53 leading to an increase of p53-MDM2 interaction, which on turn induces poliubiquitination and degradation of p53.

An evidence that suggests the ability of GRK5 to promote tumorigenesis, is its ability to inhibits p53, participating to the regulation of genome integrity. p53 is a crucial tumor suppressor that induces cell cycle arrest or apoptosis in response to diverse stresses, and its function is regulated primarily at the level of protein stability through post-translational modifications such as phosphorylation and acetylation [69, 70]. It is well established that the misregulation of p53 level or activity compromises cellular apoptotic response and contributes to tumorigenesis [71, 72]. Chen et al have shown that GRK5 phosphorylates p53 at Thr-55 and promotes its degradation, thus inhibiting p53-mediated apoptosis both in vitro (Fig. 3), in cultured human osteosarcoma cells, and in vivo [73]. In particular, GRK5 knockout mice show abnormal p53 levels and enhanced susceptibility in response to irradiation. These findings support an essential role of GRK5 to restrict p53 and protect genomic stability under physiological and pathological conditions. Moreover, this role of GRK5 was confirmed in humans, mice and bovines indicating that, it is conserved across species. How GRK5 coordinates with other p53 regulators in response to various genotoxic stresses to maintain genomic stability remains to be further elucidated, but it is clear that GRK5 also acts as stimulator of pro-tumoral effect in the cell, representing a potential target to attenuate resistance to radiation that characterizes some types of cancer.

### E. GRK5 phosphorylates moesin

**Figure 4.**
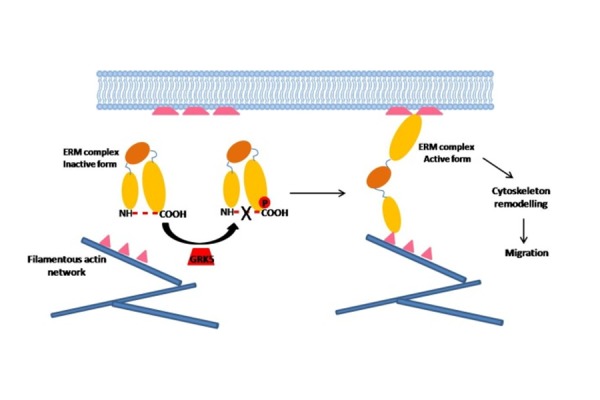
GRK5 promotes cytoskeleton remodeling inducing cancer cell migration and invasion. In the inactive form of ERM-complex, the COOH and NH domains interact, determining a close conformation of the complex. GRK5 phosphorylates an ERM-component (Moesin), interrupting this intra-molecular interaction and activating ERM-complex. In the activated form of the ERM- complex, the COOH domain interacts with cytoskeleton while the NH domain interacts with plasma membrane, inducing cytoskeleton remodeling and, consequently, cell migration and invasion.

Cancer metastasis involves the cell local invasion and migration so that detached cells from the primary tumor mass can colonize distant organs. Among the molecular mediators of cancer cell migration and invasion, moesin is part of ERM complex (Ezrin-Radixin-Moesin) that links membrane components to actin cytoskeleton, regulating cytoskeleton remodeling and cell adhesions [74, 75]. Altered expression or intracellular distribution of ERMs has been linked to tumor metastasis. In particular, ERM proteins interact with membrane through an N-terminal FERM domain, and with actin through C-terminal domain. The intra-molecular interaction between these two domains masks several binding sites leading to inactivation of ERM proteins [74]. The phosphorylation of ERM proteins by different kinases interrupts this intra-molecular interaction and activates the ERM-complex [74]. Chakraborty at al demonstrated that GRK5 colocalizes with moesin on the plasma membrane, catalyzes its phosphorylation at T66 residue, and regulates cellular distribution of moesin promoting actin remodeling and, then, invasion and metastasis of PC3 cells (Fig. 4)[76]. Moreover, in a xenograft model of human prostate cancer, GRK5 silencing reduced tumor growth, invasion and metastasis [76]. Taken together, these results propose GRK5 as a key contributor to the growth and metastasis of prostate cancer.

### F. GRK5 phosphorylates nucleophosmin, NPM1

Cancer cells are able to escape from normal mechanisms of cell cycle control; indeed, many “*oncosuppressors”* are genes that act as cell cycle – checkpoint, such as Rb and p53. These genes, determine a cell cycle arrest when the cellular genome has accumulated irreversible damages, or when the extracellular environment is not conducive to cell replications (i.e. without growth factors). Genetic alterations that determine loss of function of these genes are typical of cancer cell [77]. Among the regulator of cell cycle progression in cancer, GRK5 could represent a candidate molecule, given its nuclear localization and the identification of new nuclear substrates of this kinase. Indeed, it has been demonstrated that in the nucleus GRK5 interacts with and phosphorylates nucleophosmin (NPM1)[78], a multifunctional protein involved in the regulation of cell cycle, centrosomal duplication and apoptosis, that is overexpressed in several cancer types [79]. NPM1 function is regulated primarily through phosphorylation by PLK1, that leads to the protection from cell death [80]; Indeed, the inhibitor of PLK1, that induces apoptosis, is used in the treatment of several cancers, including esophageal cancer [81], neuroblastomas [82], and others [83]. GRK5 phosphorylates NPM1 at Ser-4, a site shared with PLK1, suggesting the possibility of an interplay between GRK5 and PLK1, in the regulation of NPM1. In particular GRK5-depleted cells were more sensitive to apoptosis induced by PLK1 inhibition, while cells with high GRK5 levels exhibited reduced sensitivity to PLK1 inhibition [78]. GRK5-dependent regulation of cell sensitivity to PLK1 inhibitors is an important finding with potential implications in combined therapies with these inhibitors. Indeed, it is likely to speculate that in subjects with higher levels of GRK5, the administration of PLK1 inhibitor could be ineffective and that therapeutic approach based on both PLK1 and GRK5 inhibition, could be more effective than PLK1 inhibition alone, in certain cancer types characterized by hyperactivation of PLK1- signaling.

Several other reports support the association between GRK5 activity and cell cycle regulation. In particular in Hela cells, breast cancer cells and also in non-trasformated cells, GRK5 localizes in centrosomes during interphase and promotes G2/M transition thus affecting cell cycle progression [47]. The knock down of GRK5 leads to G2/M arrest, even if the mechanism through which GRK5 exerts this effect is not clear yet.

## IV. TARGETING GRK5 AS POTENTIAL THERAPEUTIC STRATEGY FOR CANCER

The regulation of the expression and activity of GRKs has yielded promising results in the treatment of multiple diseases, from heart failure and diabetes to cancer and inflammatory diseases, in several animal models and cell culture systems [17, 25, 84, 85]. Given these findings, a selective inhibitor of GRK2 has been synthesized and tested both in cardiovascular diseases and tumors [18, 86–88]. On the opposite, whereas its inhibition strongly correlates with cardiac protection or regression of some tumors, to date no available specific inhibitors have been designed and synthesized for GRK5. To date, only one inhibitor of GRK5 has been recently reported [89], the amlexanox, that directly binds the active site of the kinase in a manner that mimics the adenine ring of ATP, and significantly inhibits MEF2 transcriptional activity, in association with the inhibition of GRK5 in cells. However, this inhibitor is not strictly selective for GRK5, and it has not been tested in cancer yet. It could be useful to synthesize specific inhibitor of GRK5 for the treatment of those tumors characterized, for instance, by low levels of the pro-apoptotic protein p53, to promote cell cycle arrest and apoptosis.

On the opposite, given the anti-tumoral effect of GRK5 acting on GPCRs, the induction of GRK5 levels could be the effective strategy for the treatment of GPCR-dependent tumors. Several compounds are available that induce CREB activity [90], a transcription factor that regulate the expression of several genes, including GRK5 gene. Thus the use of these compounds could be effective to induce GRK5 expression in cancer.

Little is known about the nuclear effects of GRK5 in cancer. It is likely that GRK5 could enter the nucleus and regulate the activity of transcription factors which on turn induce the expression of proand anti-apoptotic genes. It has been shown that GRK5 inhibits HDAC5 in the nucleus, promoting MEF2 expression in cardiac myocytes [22, 42]. Given the main role of this enzymes in cancer progression and the use of HDAC inhibitors as anti-cancer drugs [91, 92], it could be interesting to evaluate such phenomenon also in tumor cells.

An innovative strategy has been proposed that is specific for cancer and is based on a competitive interaction with GRK5 cytosolic substrates rather than inhibition of its activity, the TAT-RH peptide [93]. TAT-RH binds IκBα, the inhibitory protein of NFκB, thus stabilizing IκBα– NFκB complex and blocking NFκB transcriptional activity. In cultured tumor cells, different dosages of TAT-RH reduced cell survival and increased apoptosis. In BALB/c mice, the anti-proliferative effects of TAT-RH appear to be dose-dependent and highest dose completely inhibits tumor growth. Thus, TAT-RH is a promising compound for the treatment of those tumors which are NFκB dependent [94].

## V. CONCLUSIONS AND FUTURE PROSPECTIVE

GRK5 is a multifunctional protein that is able to move within the cell in response to various stimuli to regulate key intracellular signaling from receptor activation, on plasmamembrane, to gene transcription, in the nucleus. Thus, GRK5 is involved in the development and progression of several pathological conditions such as cardiac hypertrophy and failure, diabetes and cancer.

The proof of concept that GRK5 is involved in the regulation of cancer progression derives from the discovery that GRK5 was part of a subset of gene targets required for mitotic progression in human cancer cells. However, its role in tumor growth is still complex and ambiguous, since GRK5 exerts opposite effects, depending on tumor cell type and kinase localization within the cell. Indeed, when the kinase is localized at plasma membrane, it often exerts an anti-tumoral effect, attenuating growth-associated pathway through its ability to desensitize GPCR and non GPCR-receptors (TSH, PGE2R, PDGFR). On the contrary, when GRK5 moves to others compartments, such as cytosol and nucleus, it promotes tumor growth acting on non receptor-substrates, such as p53, AUKA and NPM1.

Given these different effects of GRK5 on tumor growth, targeting GRK5 could be an useful anti-cancer strategy, for specific tumor types. In this context, further studies will be needed to better define the nuclear effects of GRK5 and the possibility to regulate its subcellular localization in order to regulate its functions within the tumor cell as necessary.
